# Human Error Analysis and Modeling of Medication-Related Adverse Events in Taiwan Using the Human Factors Analysis and Classification System and Logistic Regression

**DOI:** 10.3390/healthcare11142063

**Published:** 2023-07-19

**Authors:** Shu-Huan Ko, Min-Chih Hsieh, Run-Feng Huang

**Affiliations:** 1 Department of Marketing and Logistics Management, Vanung University, Taoyuan 320313, Taiwan; shuhuanko@mail.vnu.edu.tw; 2 Department of Industrial Engineering, University of Shanghai for Science and Technology, Shanghai 200093, China

**Keywords:** medication adverse event, human errors, Human Factors Analysis and Classification System, logistic regression, patient safety

## Abstract

Medical institutions worldwide strive to avoid adverse medical events, including adverse medication-related events. However, studies on the comprehensive analysis of medication-related adverse events are limited. Therefore, we aimed to identify the error factors contributing to medication-related adverse events using the Human Factors Analysis and Classification System (HFACS) and to develop error models through logistic regression. These models calculate the probability of a medication-related adverse event when a healthcare system defect occurs. Seven experts with at least 12 years of work experience (four nurses and three pharmacists) were recruited to analyze thirty-seven medication-related adverse events. The findings indicate that decision errors, physical/mental limitations, failure to correct problems, and organizational processes were the four factors that most frequently contributed to errors at the four levels of the HFACS. Seven error models of two types (error occurrence and error analysis pathways) were established using logistic regression models, and the relative probabilities of failure factor occurrences were calculated. Based on our results, medical staff can use the error models as a new analytical approach to improve and prevent adverse medication events, thereby improving patient safety.

## 1. Introduction

### 1.1. Medication Errors

Despite the progress in technology and society, patient safety remains a serious issue. However, the number of injuries or deaths caused by medical-related accidents, including medication errors, has decreased considerably [1,2]. According to Kepner and Jones, who analyzed 288,882 serious medical events from the Pennsylvania Patient Safety Reporting System, the largest repository of patient safety data in the United States, medication errors accounted for nearly 20% of medical adverse events in 2021 [3]. Similarly, a report from the Taiwan Patient Safety Reporting System (TPR System) indicated that medication errors accounted for approximately 32% of all medical adverse events in 2020 [4]. Medication errors can lead to adverse events that endanger patient safety, contribute to grievances between patients and healthcare providers [5], and increase healthcare expenses [6]. Therefore, reducing the occurrence rate of medication errors is a pressing issue in modern society.

Medication errors, defined as “a failure in the treatment process that leads to, or has the potential to harm the patient” [7], are preventable events that can occur at any step during the medication process, including prescribing, administering, and monitoring medication [5,8,9]. In Taiwan, human error is a major cause of medication errors, accounting for approximately 63.8% of all medication-related adverse events in 2020 [4]. To prevent the recurrence of human errors, analyzing medication-related adverse events to understand the deficiencies in the healthcare system and helping medical staff improve medication errors more effectively is necessary. Previous studies have analyzed and classified human error factors based on practical medication-related adverse events. Naserallah et al. conducted a systematic analysis and review of the past literature and identified the three most common errors in pediatrics: inappropriate dosing, wrong drug, and drug interaction in medication-related adverse events [9]. Pham et al. reported that the most frequent medication errors were inappropriate doses in the emergency department, whereas failure to follow procedure and poor communication were the top two leading causes among medical staff [6]. Other types of medication errors, such as the label used for drugs, communication capabilities of medical staff, timing of drug administration, and interruption, were also identified in some studies [5,10,11,12]. Psychological mechanisms have also been considered to analyze and classify medication-related adverse events; distractions, increased workload, and violations are typical error factors that can contribute to medication-related adverse events [6,12,13].

Medication errors are usually caused by disparate and complex factors [5] that do not occur in a single unsafe condition [14,15]. Cigularov et al. reported that the safety awareness of employees in an organization stems from a good safety culture that could decrease the number of errors [16]. If an organization does not develop safety rules, regulations, and culture, employees’ safety awareness will be insufficient, increasing the probability of an error [17]. Therefore, when conducting an error analysis, analyzing the unsafe behaviors of humans that cause the error and exploring the underlying causes to understand the error context better and prevent future similar incidents is important [2].

### 1.2. Human Factors Analysis and Classification System (HFACS)

With the increased amount of research on human errors, several human error analysis methods and classification models have been developed, including the HFACS, SHELL model, root cause analysis, human error assessment and reduction technique, the technique for the retrospective and predictive analysis of cognitive errors, and systematic human error reduction and prediction approach. Compared with other methods, HFACS provides a relatively comprehensive analysis framework that considers behavioral, managerial, and organizational factors and is widely used for accident analysis in aviation safety [1,18,19,20,21], patient safety [2,22], railway and marine safety [23,24], the oil and gas industry [25], and other fields. The HFACS was developed by Wiegmann and Shappell (2012) based on the Swiss cheese model [14], which provides a relatively complete structural framework for human error analysis [26]. The HFACS divides error factors into four main categories: unsafe acts (level 1), preconditions for unsafe acts (level 2), unsafe supervision (level 3), and organizational influences (level 4). Unsafe acts represent medical staff behavior that directly or indirectly contributes to medication-related adverse event occurrence during medical practice, such as decision errors and skill-based errors. Preconditions for unsafe acts refer to the potential causes of unsafe acts, including physiological, psychological, and environmental issues, such as adverse mental states and physical/mental limitations. Unsafe supervision indicates supervision problems in medication-related adverse events, such as inadequate supervision or failure to correct problems, which may lead to preconditions for unsafe acts. Organizational influences represent the problems within the healthcare systems that affect supervision and personnel behavior, often perceived as the root cause of medication-related adverse events, such as resource management and organizational climate.

The concept of the HFACS is that error factors at higher levels can influence or cause errors at lower levels, providing medical staff with a comprehensive understanding of the occurrence of adverse medication events to make the necessary improvements. In addition, by analyzing the causality between these error factors, medical staff can understand the characteristics of medication-related adverse events and prevent errors, thereby reducing medication-related adverse events and improving patient safety. Therefore, exploring the causality between the different levels of error factors is necessary.

### 1.3. Causality between Error Factors

Understanding and analyzing the causality of error factors is important. For example, in hospitals, younger medical staff often schedule their days off on consecutive days, leading to extended duty periods without rest, which can cause incorrect decisions or inappropriate behaviors. Such errors can lead to adverse medical events and cause great harm to patients. However, the day-off schedule of medical staff is reviewed by supervisors who do not detect such poor scheduling, indicating a hazardous supervision process. Error factors are interrelated, and their causality should be explored to help medical staff to better understand the antecedents and consequences of adverse events. Previous studies have applied the method known as the odds ratio to calculate the strength of association between two factors. Hsieh et al. used HFACS to analyze medication-related adverse events and to identify the causality between the error factors using the odds ratio [26]. Naseralallah et al. also used the odds ratio to calculate the rate of medication errors [9]. Both studies determined whether the relationship between different error factors was strong or weak. Hsieh et al. revealed that the frequency of error factors might be underestimated if the causality between error factors is not considered. In other words, when the causality between error factors is well understood, the frequency of error factors can be calculated more accurately, which provides a prediction method for their occurrence [27].

Regarding error prediction, Xie and Guo indicated that human errors involve uncertainties [1]. Thus, the Markov chain can be used to predict human error factors. Markov chains generate a one-step transition matrix based on conditional probability and represent the regularity of an event after an n-step transformation, assuming that the next state of an event is related only to the current state and not to the historical state. Therefore, regardless of the stage at which the event occurs, the Markov chain can successfully predict the state change in the event in the next stage, which is commonly used in the reliability assessments of power systems and equipment [28]. However, the Markov chain concept is inconsistent with the HFACS. When analyzing human error, researchers must attempt to determine the latent error factors (error factors that cause accidents indirectly) [20] behind accidents and identify the root cause. This is important in terms of medication safety because it helps researchers comprehensively understand accidents and facilitates subsequent improvements. In other words, in human error analysis, the next state of the error factors is related to the current state and the historical state. Therefore, conditional probability and the Markov chain may be unsuitable for comprehensively analyzing medication-related adverse event occurrence in our study.

A specific mathematical tool is required to develop a model that relates the current and historical states of medication-related adverse events to predict future states of error development. In recent years, logistic regression has been used in many studies to predict and prevent accidents in different fields, such as aviation [29], tank farms [30], ship collisions [31], and petrochemical industries [32]. Many studies have analyzed the error factors of medication-related adverse events; however, few have considered the present and historical states of the lapse factors when calculating the occurrence frequency of error factors. The frequency of error factors may be underestimated when predicting error factors, which affects the subsequent prevention and improvement of medication-related adverse events. In addition, when performing error analysis with the HFACS, each error factor is coded in binary (1 for presence and 0 for absence). In accordance with the data characteristics, we used logistic regression to explore the causality between each error factor [32].

### 1.4. The Purposes of this Study

First, we aimed to analyze medication-related adverse events using the HFACS framework and identify the latent error factors to understand the causes of medication-related adverse events. Second, we aimed to develop mathematical models for predicting human errors using logistic regression and to determine the causality between the error factors. Both analytical methods can provide a comprehensive review of medication-related adverse events, which can serve as a reference for medical staff in education, training, adverse event analysis, and the development of improvement strategies to enhance patient safety.

## 2. Materials and Methods

We conducted a retrospective study on human error analysis and modeling of historical data for medication-related adverse events. The HFACS framework was used to classify and analyze human error factors in medication-related adverse events. The error analysis results were modeled using logistic regression to present the causality between error factors. Thus, the occurrence frequency of the error factors can be determined using the models in this study. This study was approved by the Institutional Review Board (approval number: 14MMHIS212) of our hospital.

### 2.1. Selection of Medication-Related Adverse Events

To provide a comprehensive analysis of medication-related adverse events, this study was conducted in collaboration with a regional hospital in Hsinchu, Taiwan, which has more than 400 beds. The hospital provided 12 near-miss medication-related adverse events between 1 January 2019 and 31 December 2021 for this study to analyze human errors. We also screened appropriate cases from the TPR system to increase the number and diversity of medication-related adverse events, which include various medical adverse events, such as medication errors and falls.

We used the TPR system, which is an open system under the joint commission of Taiwan’s medical facilities. The main purpose of the system is to allow medical institutions throughout Taiwan to voluntarily and anonymously upload medical adverse events that occur in hospitals and then provide statistical information related to patient safety on a quarterly basis. In addition, the system also holds regular educational training so as to enhance the safety of patients in Taiwan. We screened the TPR system cases in two steps. First, cases not related to medication were excluded. Second, we further screened cases from the TPR system because they were from many medical institutions, and their medications and administration procedures differed from those used in the collaborating hospital. For the two steps of case screening, two medical staff members with at least 10 years of medical experience in the collaborating hospital were recruited from the pharmacy and nursing departments (four in total, three women and one man). These two steps ensured that the cases included in this study were all medication-related adverse events related to the collaborating hospital.

### 2.2. Medication Error Analysis

The analysis of medication-related adverse events requires years of experience working in the same hospital so that one can understand the hospital rules and systems well enough to facilitate the analysis of medication-related adverse events. Thus, seven medical staff with more than 12 years of work experience were recruited from the nursing and pharmacy departments of the collaborating hospital (excluding the four medical staff who participated in the case-screening phases). Of these, four were from the nursing department (including one vice director), and three were from the pharmacy department (including one technical director). None of the seven medical staff members had knowledge of the HFACS; therefore, appropriate educational training was required to ensure the validity of the results before performing the error analysis. We conducted at least 10 h of training for the seven medical staff on applying the HFACS framework to analyze medication-related adverse events; two additional cases were used to confirm their learning performance. The charting of events and causal factors, a method used by the Department of Energy, United States [33], was applied to transform the cases from word descriptions to flowcharts. The flowchart included each medical action in the cases (for example, prescribing a medical order or injection) and the time of the action to assist medical staff in clarifying the medication-related adverse events that occurred and accurately determine the latent error factors [26].

**Figure 1:**
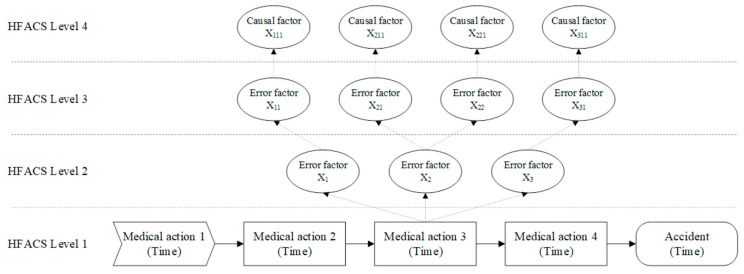
The schematic of the error analysis process.

Before the formal analysis, the medical staff read, discussed, and confirmed the details of the medication-related adverse events. After 10–15 min of discussion, they used flowcharts to identify the key medical actions that potentially influenced the occurrence of medication-related adverse events and categorized them into one of the four error factors in level 1 of the HFACS. Due to the retrospective study design, we could not fully reconstruct the medication-related adverse events through descriptions alone. Therefore, during the analysis process, the medical staff identified as many potential key medical actions as possible, which allowed for the identification of several key medical actions in a single medication-related adverse event. Subsequently, further analysis was conducted for each key medical action according to the HFACS framework (Figure 1). When analyzing medication-related adverse events, an error factor of 1 was recorded for those that occurred in the adverse event and 0 for the rest. After analyzing one case, at least two error paths from level 4 to level 1 were identified, and the paths were recorded.

### 2.3. Logistic Regression

Logistic regression is an analytic method used to establish probabilistic models for predicting future events by fitting accident systems [29,34]. The mathematical presentation of the logistic regression model based on the generalized linear model is presented in Equation (1). Equation (2) is used to determine the logic of the logistic regression model, and the logistic regression model is represented in the following Equation (3) [35]:

(1) $$P\left(X\right)=\frac{e^{\beta_{0}+\beta_{1}X_{1}+\cdots +\beta_{n}X_{n}}}{e^{\beta_{0}+\beta_{1}X_{1}+\cdots +\beta_{n}X_{n}}+1}$$

(2) $$g\left(X\right)=\beta_{0}+\beta_{1}X_{1}+\cdots +\beta_{n}X_{n}$$

(3) $$P\left(X\right)=\frac{e^{g\left(X\right)}}{e^{g\left(X\right)}+1}$$

where $X=X_{1},\;X_{2},\cdots ,\;X_{n}$ are predictors that represent the error factors in this study.

Through logistic regression analysis, we established equations for predicting the error factors, which may be used to predict the occurrence frequency of error factors and to comprehensively understand medication-related adverse events.

## 3. Results

### 3.1. Medication-Related Adverse Event Analysis

In total, 175 adverse medical events were listed in the TPR system as learning cases. Of these, 53 were identified as medication-related adverse events based on the first step of the case screening. Of the 53 medication-related adverse events, 25 were identified as events that might occur in the collaborating hospital. Therefore, 37 cases (12 from the collaborating hospital) related to medication errors were included in this study. After error analysis, 248 paths were identified. The seven medical staff members identified the error factors, and the number of error factors was recorded to calculate the occurrence rate and to implement the logistic regression analysis. Table 1 presents the occurrence rate and number of error factors for the HFACS.

### 3.2. Logistic Regression Analysis

Logistic regression analysis was applied to determine the causality of the error factors among HFACS levels 4, 3, 2, and 1. We analyzed the error pathways in two directions: from level 4 to level 1 (top-down) and from level 1 to level 4 (bottom-up). The top-down error pathway is the pathway through which errors occur, which can be used to understand the likelihood of an adverse event and prevent it when a defect in the healthcare system is identified. The dependent variable, the occurrence rate, was divided into four groups based on the error factors in HFACS level 1, where P_D_, P_S_, P_P_, and P_V_ represent the occurrence rates of decision errors, skill-based errors, perceptual errors, and violations, respectively. The independent variables were the error facts of the HFACS at levels 2, 3, and 4. The equations for the top-down error pathway are as follows:

(4) $$P_{D}=\frac{e^{1.556-1.139X_{ps}-1.126X_{cc}-1.393X_{l}-1.987X_{ms}+0.523X_{sv}-0.266X_{fc}+0.021X_{pi}-0.097X_{op}-0.316X_{oc}}}{e^{1.556-1.139X_{ps}-1.126X_{cc}-1.393X_{l}-1.987X_{ms}+0.523X_{sv}-0.266X_{fc}+0.021X_{pi}-0.097X_{op}-0.316X_{oc}}+1}$$

(5) $$P_{S}=\frac{e^{-3.009+0.875X_{ps}+1.422X_{cc}-0.995X_{l}+0.988X_{ms}-20.637X_{sv}-0.305X_{fc}-0.295X_{pi}+1.413X_{op}-2.183X_{oc}}}{e^{-3.009+0.875X_{ps}+1.422X_{cc}-0.995X_{l}+0.988X_{ms}-20.637X_{sv}-0.305X_{fc}-0.295X_{pi}+1.413X_{op}-2.183X_{oc}}+1}$$

(6) $$P_{P}=\frac{e^{-22.49+20.162X_{ps}+1.473X_{cc}+18.249X_{l}+20.089X_{ms}-17.504X_{sv}+1.843X_{fc}+1.425X_{pi}-0.461X_{op}-1.426X_{oc}}}{e^{-22.49+20.162X_{ps}+1.473X_{cc}+18.249X_{l}+20.089X_{ms}-17.504X_{sv}+1.843X_{fc}+1.425X_{pi}-0.461X_{op}-1.426X_{oc}}+1}$$

(7) $$P_{V}=\frac{e^{-2.045-19.077X_{ps}-19.158X_{cc}+1.87X_{l}+0.774X_{ms}+1.453X_{sv}+0.033X_{fc}-0.143X_{pi}-0.306X_{op}-0.354X_{oc}}}{e^{-2.045-19.077X_{ps}-19.158X_{cc}+1.87X_{l}+0.774X_{ms}+1.453X_{sv}+0.033X_{fc}-0.143X_{pi}-0.306X_{op}-0.354X_{oc}}+1}$$

**Figure 2:**
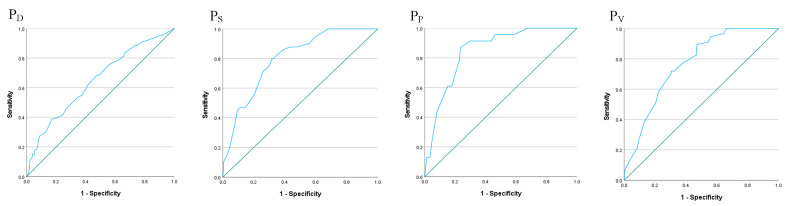
The AUC-ROC curves for the top-down error pathway.

Using a top-down error pathway (organizational climate → planned inappropriate operations → adverse mental states) as an example, the estimated occurrence rates for decision errors, skill-based errors, perceptual errors, and violations were calculated using Equations (4)–(7). In the top-down error pathway, the occurrence rates of decision errors, skill-based errors, perceptual errors, and violations were 32.6 %, 46.6 %, 8.3%, and 14.5%, respectively. Based on logistic regression analysis, the precision of the estimated occurrence rate for error factors, including decision errors, skill-based errors, perceptual errors, and violations, was 58.9%, 80.6%, 90.7%, and 78.2%, respectively. The Hosmer–Lemeshow test was applied to test the goodness-of-fit (GOF) of the equations. The GOF results indicated that Equations (4)–(7) fit the observed data well (the *p*-values in Equations (4)–(7) were 0.946, 0.403, 0.610, and 0.995, respectively). The predictive accuracy of the model was additionally evaluated using AUC-ROC curves (Figure 2). The results showed that the AUC test values for Equations (4)–(7) were 0.647, 0.799, 0.848, and 0.767, respectively. Six top-down error pathways were randomly selected as examples to illustrate the different occurrence rates of error factors in HFACS level 1, as presented in Table 2.

The bottom-up error pathway is the path of error analysis, where the type of error that occurred is used to infer other error factors that may exist in the healthcare system and quantify the possibility of their existence. They are used for post-incident estimation and improvement. In the bottom-up error pathway analysis, the dependent variable, the occurrence rate, was divided into three groups based on the error factors in HFACS level 4; P_RM_, P_OC_, and P_OP_ represent the occurrence rates of resource management, organizational climate, and organizational process, respectively. The independent variables were the HFACS level 1, 2, and 3 error factors. The equations for the bottom-up error pathway are as follows:

(8) $$P_{RM}=\frac{e^{0.094-1.443X_{s}+0.592X_{p}+0.241X_{v}-1.255X_{ms}-1.069X_{l}-2.216X_{cc}-0.439X_{ps}+0.584X_{pi}-1.219X_{fc}-1.034X_{sv}}}{e^{0.094-1.443X_{s}+0.592X_{p}+0.241X_{v}-1.255X_{ms}-1.069X_{l}-2.216X_{cc}-0.439X_{ps}+0.584X_{pi}-1.219X_{fc}-1.034X_{sv}}+1}$$

(9) $$P_{OC}=\frac{e^{-1.265+0.865X_{s}-0.869X_{p}-0.032X_{v}+0.414X_{ms}+0.27X_{l}+1.554X_{cc}+0.667X_{ps}-1.369X_{pi}+0.221X_{fc}-0.236X_{sv}}}{e^{-1.265+0.865X_{s}-0.869X_{p}-0.032X_{v}+0.414X_{ms}+0.27X_{l}+1.554X_{cc}+0.667X_{ps}-1.369X_{pi}+0.221X_{fc}-0.236X_{sv}}+1}$$

(10) $$P_{OP}=\frac{e^{-0.476-0.122X_{s}+0.092X_{p}-0.113X_{v}+0.246X_{ms}+0.292X_{l}-0.527X_{cc}-0.302X_{ps}+0.342X_{pi}+0.429X_{fc}+0.827X_{sv}}}{e^{-0.476-0.122X_{s}+0.092X_{p}-0.113X_{v}+0.246X_{ms}+0.292X_{l}-0.527X_{cc}-0.302X_{ps}+0.342X_{pi}+0.429X_{fc}+0.827X_{sv}}+1}$$

**Figure 3:**
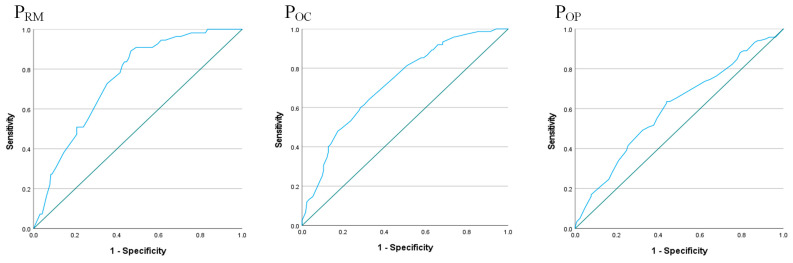
The AUC-ROC curves for the bottom-up error pathway.

Using a bottom-up error pathway (skill-based errors → adverse physiological states, and → planned inappropriate operations) as an example, the estimated occurrence rates for resource management, organizational climate, and organizational processes were calculated using Equations (8)–(10), respectively. In the bottom-up error pathway, the occurrence rates for resource management, organizational climate, and organizational process were 23.0%, 24.9%, and 36.4%, respectively. From the logistic regression analysis, the precision of the estimated occurrence rate for error factors, including resource management, organizational climate, and organizational process, was 76.6%, 71.8%, and 58.1%, respectively. The Hosmer–Lemeshow test was applied to test the GOF of the equations in this study. The GOF results indicated that Equations (8)–(10) fit the observed data well (the *p*-values in Equations (8)–(10) were 0.613, 0.767, and 0.855, respectively). The AUC-ROC curve was used to assess the predictive accuracy of the model (Figure 3), and the results showed that the AUC test values of Equations (8)–(10) were 0.603, 0.723, and 0.745, respectively. Six bottom-up error pathways were randomly selected in this study as examples to illustrate the different occurrence rates of error factors in HFACS level 4, as presented in Table 3.

## 4. Discussion

We analyzed 37 medication-related adverse events that occurred in the collaborating hospital through the HFACS. Decision errors, physical/mental limitations, failure to correct problems, and organizational processes were the most frequent error factors in the four levels of the HFACS, with incidence rates of 47.98%, 37.1%, 39.52%, and 47.58%, respectively. Compared with previous studies [5,12], this study covers a wider range of error factors, allowing researchers to understand the causes of adverse events more easily. The HFACS framework used in this study contains both active human errors (resulting in accidents directly; their influence is immediate, which is also recorded in the general accident report) and latent human errors (error factors that cause accidents indirectly) [20]. Regarding active human error, actions that directly led to adverse events were recorded, such as medication-related adverse events caused by medical staff who did not perform the checking procedure. Previous studies have focused more on active human errors and have paid less attention to the latent errors (causes of these unsafe behaviors, such as management- and organization-related issues), potentially leading to proposed improvements that only treat the symptoms and not the root cause. The present study complements the shortcomings of previous studies. Briefly, this study included active and latent error factors, which made it easier for medical staff to understand the problems hidden in the healthcare system and to focus on them for improvement.

In addition to analyzing the active and latent error factors, we analyzed two types of error factor occurrence rates through logistic regression: the error occurrence pathway (top-down) and error analysis pathway (bottom-up), and developed a model related to medication human errors. Seven human error models were developed to analyze the occurrence rate of error factors based on level 1 (active error factors) and level 4 (latent error factors), while other error factors (levels 2 and 3) occurred. From the perspective of the “error occurrence pathway”, medical staff can understand when a specific error factor occurs. For example, when the error pathway in Table 2 occurs (organizational climate → planned inappropriate operations → adverse mental states), Equations (4)–(7) reveal that the occurrence rates of each error factor in level 1 of the HFACS are 0.362 (decision errors), 0.466 (skill-based errors), 0.083 (perceptual errors), and 0.195 (violations), which are particularly important for education and training within medical institutions. During general routine educational training, in addition to acquiring new medical knowledge and skills, recent adverse medical events should be discussed. However, most studies have focused on the technical problems that occurred in an event and rarely reviewed the deficiencies related to management or the correlation between the deficiencies. Through the medical human error model related to the “error occurrence pathway” proposed in this study, medical staff can not only be more aware of some unreasonable systems and behaviors in their daily work but also realize that otherwise perceived unimportant issues, such as nursing staff scheduling, can cause unnecessary medical errors, thus, improving their behavior in their daily nursing work to enhance patient safety.

When an adverse event occurs at a medical institution, the focus is usually only on the surface phenomena for discussion and improvement. For example, if a medical staff member misreads a patient’s name and bed number and causes a medication incident, a double-check action is added to the medication administration process during the review meeting to prevent similar situations from recurring. However, adding additional actions to the existing operation process will likely burden the medical staff, causing other problems, an example of treating the symptoms but not the root cause. Thus, for error models based on “error analysis pathways”, healthcare professionals can more easily identify the factors that cause current errors in medical institutions. For instance, when the error pathway in Table 3 occurs (skill-based errors → adverse mental states → planned inappropriate operations), the probabilities of each error factor in level 4 are 0.205 (organizational climate), 0.117 (resource management), and 0.497 (organizational process) through error models (8)–(10). This information can help medical staff better understand potential problems in the healthcare system and improve the whole system.

Previous studies have investigated the factors contributing to adverse medical events and compiled the probability of their occurrence. For example, inappropriate dosing, wrong drugs, labels used for drugs, communication capabilities of medical staff, distractions, increasing workload, and violations [5,6,9,12] are all error factors that have a high probability of occurrence in healthcare systems. However, error factors are correlated [14]; thus, Hsieh et al. used the HFACS as an analytical framework to present the relationship between factors of two adjacent levels through conditional probability [27]. In their study, they explained the probability of a particular error affecting the occurrence of the following errors. Our study is similar to Hsieh et al. in that we used HFACS as the analytical framework; however, we additionally considered the concepts of “error occurrence pathway” and “error analysis pathway” and built a failure model through logistic regression to provide medical staff with a more comprehensive and in-depth understanding of the problems that may be hidden in the organization and management modality when a specific failure path occurs (based on error occurrence pathway), or the types of failures that may occur (based on error analysis pathway), and to prevent them from occurring. Generally, this study considers the correlation and predictability among the failure factors, and the proposed model somewhat fills the gap in this part of the previous studies, which adds depth and breadth to the field of medical human error analysis.

This study had three limitations. First, the model developed in this study was based on cases in the collaborating hospital; thus, it only applies to the collaborating hospital. Therefore, our results may not represent all adverse drug events in Taiwanese hospitals. Second, previous studies have provided more detailed descriptions of failure factors, such as inappropriate dosing, wrong drugs, and increasing workloads. However, the HFACS framework used in this study did not include the details of the actions, which is a direction for future research. Lastly, this was a retrospective study, and it was difficult to reflect the real situation during the medication-related adverse event through case studies; therefore, the results may be slightly inaccurate.

## 5. Conclusions

We analyzed human error factors in medication-related adverse events using HFACS and established a model of error pathways using logistic regression. Decision errors, physical/mental limitations, failure to correct problems, and organizational processes were the four factors with the highest occurrence of errors at the four levels of the HFACS. We also constructed two types of failure models based on the analyzed failure paths: the error occurrence pathway (top-down) and the error analysis pathway (bottom-up). Based on our results, an error model based on an error occurrence pathway can help medical staff understand the types of human errors caused by inappropriate systems and management modalities during education and training. Furthermore, an error model based on the error analysis pathway can help medical staff rapidly conduct a preliminary analysis of medication-related adverse events when they occur and determine the probability of each potential error factor, which can improve the efficiency of problem detection.

Improving patient safety is a universal goal of medical practitioners; therefore, they strive to reduce medical negligence and the resulting harm. The research results are only suitable for application to the collaborating hospital; however, the analysis process and modeling method can be used as references for other medical institutions. By exploring medical adverse events in hospitals to build an error model, medical staff can further understand that any seemingly minor problem may affect patient safety in the future and allow decision-makers to develop a comprehensive system to improve the operation of medical institutions. This study was conducted on medication-related adverse events in only one medical institution; therefore, researchers should integrate drug events in different medical institutions and elevate the perspective from a single medical institution to the entire regional or national medical environment, and then formulate medical-related policies to improve the overall medical environment and enhance patient safety.

## Figures and Tables

**Table 1:** The number and occurrence rate of error factors in medication-related adverse events (n = 248).

Error Factors of HFACS (Acronym)	Number of Errors	Occurrence Rate of Error Factors
*Unsafe acts*		
Decision errors (d)	119	47.98%
Skill-based errors (s)	49	19.76%
Perceptual errors (p)	23	9.27%
Violations (v)	57	22.98%
*Preconditions for unsafe acts*		
Technological environment (te)	21	8.47%
Adverse mental states (ms)	86	34.68%
Physical/mental limitations (l)	92	37.10%
Communication, coordination, planning (cc)	22	8.87%
Adverse physiological states (ps)	27	10.89%
*Unsafe supervision*		
Inadequate supervision (is)	75	30.24%
Planned inappropriate operations (pi)	58	23.39%
Failure to correct problem (fc)	98	39.52%
Supervisory violations (sv)	17	6.85%
*Organizational influence*		
Resource management (rm)	55	22.18%
Organizational climate (oc)	75	30.24%
Organizational process (op)	118	47.58%

Abbreviations: HFACS, Human Factors Analysis and Classification System.

**Table 2:** Examples of the top-down error pathway for the occurrence rate of error factors in HFACS level 1.

The Error Factors of HFACS from Level 4 to Level 2			The Occurrence Rate of Error Factors in Level 1 of HFACS			
Level 4	Level 3	Level 2	Decision Errors	Skill-Based Errors	Perceptual Errors	Violations
Organizational climate	Planned inappropriate operations	Adverse mental states	0.326	0.466	0.083	0.195
	Failure to correct problem	Adverse physiological states	0.458	0.436	0.128	0.000
Resource management	Planned inappropriate operations	Adverse mental states	0.398	0.089	0.273	0.196
	Failure to correct problem	Adverse physiological states	0.537	0.08	0.381	0.000
Organizational process	Planned inappropriate operations	Adverse mental states	0.375	0.288	0.192	0.151
	Failure to correct problem	Adverse physiological states	0.513	0.263	0.279	0.000

**Table 3:** Examples of the bottom-up error pathway for the occurrence rate of error factors in HFACS level 4.

The Error Factors of HFACS from Level 1 to Level 3			The Occurrence Rate of Error Factors in Level 4 of HFACS		
Level 1	Level 2	Level 3	Organizational Climate	Resource Management	Organizational Process
Skill-based errors	Adverse mental states	Planned inappropriate operations	0.205	0.117	0.497
	Physical/mental limitations	Supervisory violations	0.409	0.030	0.627
Perceptual errors	Adverse mental states	Planned inappropriate operations	0.043	0.503	0.551
	Physical/mental limitations	Supervisory violations	0.109	0.195	0.676
Decision errors	Adverse mental states	Planned inappropriate operations	0.097	0.359	0.527
	Physical/mental limitations	Supervisory violations	0.226	0.118	0.655

## Data Availability

The data presented in this study are available on request from the corresponding author. The data are not publicly available due to privacy reasons.
